# Presence of Human Papillomavirus and Epstein–Barr Virus, but Absence of Merkel Cell Polyomavirus, in Head and Neck Cancer of Non-Smokers and Non-Drinkers

**DOI:** 10.3389/fonc.2020.560434

**Published:** 2021-01-20

**Authors:** Frans J. Mulder, Faisal Klufah, Famke M. E. Janssen, Farzaneh Farshadpour, Stefan M. Willems, Remco de Bree, Axel zur Hausen, Mari F. C. M. van den Hout, Bernd Kremer, Ernst-Jan M. Speel

**Affiliations:** ^1^ Department of Otorhinolaryngology and Head & Neck Surgery, GROW-School for Oncology and Developmental Biology, Maastricht University Medical Center, Maastricht, Netherlands; ^2^ Department of Pathology, GROW-School for Oncology and Developmental Biology, Maastricht University Medical Center, Maastricht, Netherlands; ^3^ Department of Laboratory Medicine, Faculty of Applied Medical Sciences, Albaha University, Albaha, Saudi Arabia; ^4^ Department of Otorhinolaryngology, BovenIJ Hospital, Amsterdam, Netherlands; ^5^ Department of Pathology, University Medical Center Utrecht, Utrecht, Netherlands; ^6^ Department of Pathology, University Medical Center Groningen, Groningen, Netherlands; ^7^ Department of Head and Neck Surgical Oncology, University Medical Center Utrecht, Utrecht, Netherlands

**Keywords:** head and neck cancer, human papillomavirus, Epstein–Barr virus, polyomavirus, non-smokers, non-drinkers, cell cycle protein, *in situ* hybridization

## Abstract

**Objective:**

Determine the presence and prognostic value of human papillomavirus (HPV), Epstein-Barr virus (EBV), Merkel cell polyomavirus (MCPyV), and cell cycle proteins in head and neck squamous cell carcinoma (HNSCC) of non-smokers and non-drinkers (NSND).

**Methods:**

Clinical characteristics and tumors of 119 NSND with HNSCC were retrospectively collected and analyzed on tissue microarrays. RNAscope *in situ* hybridization (ISH) was used to screen for the presence of HPV and MCPyV mRNA. Immunohistochemistry was performed for expression of p16 as surrogate marker for HPV, Large T-antigen for MCPyV, and cell cycle proteins p53 and pRb. Positive virus results were confirmed with polymerase chain reaction. For EBV, EBV encoded RNA ISH was performed. Differences in 5-year survival between virus positive and negative tumors were determined by log rank analysis.

**Results:**

All oropharyngeal tumors (OPSCC) (n = 10) were HPV-positive, in addition to one oral (OSCC) and one nasopharyngeal tumor (NPSCC). The other three NPSCC were EBV-positive. MCPyV was not detected. Patients with HPV or EBV positive tumors did not have a significantly better 5-year disease free or overall survival. Over 70% of virus negative OSCC showed mutant-type p53 expression.

**Conclusion:**

In this cohort, all OPSCC and NPSCC showed HPV or EBV presence. Besides one OSCC, all other oral (n = 94), hypopharyngeal (n = 1), and laryngeal (n = 9) tumors were HPV, EBV, and MCPyV negative. This argues against a central role of these viruses in the ethiopathogenesis of tumors outside the oro- and nasopharynx in NSND. So, for the majority of NSND with virus negative OSCC, more research is needed to understand the carcinogenic mechanisms in order to consider targeted therapeutic options.

## Introduction

Viruses play an increasing role in head and neck squamous cell carcinoma (HNSCC). High-risk human papillomavirus (HPV)-positive oropharyngeal squamous cell carcinoma (OPSCC) has been identified as an entity with a different carcinogenesis than traditional HNSCC resulting from excessive tobacco and alcohol consumption. HPV is also an independent prognostic factor for a better disease free survival (DFS) and overall survival (OS), which has led to a down staging of these tumors in the eighth edition of the American Joint Committee on Cancer (AJCC) and union for International Cancer Control tumor-node-metastasis (TNM) classification (1–3). Because of the better prognosis, de-escalation strategies are proposed for HPV-positive OPSCC patients (4). The prevalence of HPV-positive OPSCC is rising in the Western World. A HPV prevalence above 50% has already been reported in America, Europe, and Australia, based on HPV DNA in combination with either E6*I mRNA or p16 immunohistochemistry (IHC) detection (5, 6). Combining these HPV detection methods has been recommended because only OPSCC with transcriptionally active HPV is related to a better survival compared to biologically inactive infections (7, 8).

Another virus known for its carcinogenic potential in the head and neck region is the Epstein-Barr virus (EBV). EBV has a strong association with nasopharyngeal squamous cell carcinoma (NPSCC), approaching a prevalence of 100% in these tumors, and is endemic in Southern China, Southeast Asia, Northern Africa, and the Mediterranean basin (9, 10). It is suggested to cause an immunosuppressive microenvironment in these tumors, among others *via* PD-L1 overexpression, making these patients interesting candidates for checkpoint blockade therapy (10). Detection of EBV presence can be performed reliably with EBV encoded RNA (EBER) *in situ* hybridization (ISH) (9, 11).

Lately, besides these acknowledged oncogenic viruses, there is attention for polyomaviruses in HNSCC. Merkel cell polyomavirus (MCPyV) has not only been detected by digital transcriptome subtraction and polymerase chain reaction (PCR) in up to 80% of Merkel cell carcinoma of the skin, but also in non-malignant tonsillar tissue, oral squamous cell carcinoma (OSCC), and pharyngeal cancer, with a reported prevalence of 23, 6.6–29, and 50% respectively (12–17). Although it was thought not to play a role in oral carcinogenesis because of low viral loads detected with quantitative real-time PCR, the presence of MCPyV appears to be predictive for a better DFS (16).

Cell cycle deregulation plays a central role in head and neck carcinogenesis, with frequent inactivation of *TP53* and *CDKN2A*, leading to cell proliferation and prevention of apoptosis, among others. In HPV-related OPSCC, HPV integration in the host cell DNA genome leads to deregulation of oncoproteins E6 and E7, resulting in inactivity of p53 and retinoblastoma tumor suppressor gene product pRb, respectively. The negative feedback of pRb inactivation leads to p16 overexpression (18). In EBV infected NPSCC, it has been suggested that the cell cycle pathway is the most deregulated pathway, promoting the progression of the G1/S phase *via* inhibition of p16 expression and pRb overexpression (19). For MCPyV, oncogenetic transformation requires both integration of the viral genome into the host genome and truncation of the Large T-antigen (LTAg) to render the viral genome replication deficient (20). LTAg mutations disrupt the DNA binding domain and the helicase domain distal to the pRb-binding motif, thereby promoting cell cycle progression by retaining its ability to bind to pRb (20, 21).

There is a small group of HNSCC patients without any exposure to the traditional risk factors. The mechanisms underlying carcinogenesis in these non-smokers and non-drinkers (NSND) remain largely unclear, but a significant role of oncogenic viruses would be expected. Indeed, a higher prevalence of HPV in these tumors has been reported in several studies, though in small numbers of patients (22–25). Therefore, the goal of this study was to determine the presence of HPV, EBV, and MCPyV in a series of 119 well-characterized NSND with HNSCC. Secondary analyses evaluate differences in tumor suppressor proteins p16, p53, and pRb expression regarding viral presence and whether the presence of these viruses is predictive for a better DFS and OS.

## Materials and Methods

### Patients

Consecutive patients with HNSCC were selected at the University Medical Center Utrecht (UMCU) and Maastricht University Medical Center (MUMC). In the UMCU, patients were prospectively selected between 1980 and 2004, as described previously (26). In the MUMC, HNSCC patients have been selected retrospectively between 2011 and 2016, in addition to all patients with OPSCC between 2003 and 2010. Inclusion criteria were: ≥18-years-old NSND patients with HNSCC, available formalin fixated and paraffin embedded (FFPE) tumor tissue, and >2 years follow up. Patient characteristics, risk factors, World Health Organization tumor classification, AJCC seventh edition staging, and information concerning recurrent disease or death were collected from the medical records. Non-smoking was defined as having no history of smoking, non-drinking as having no history of alcohol consumption (not even ‘sporadic’ alcohol consumption), as reported in the patients’ medical records during both their first presentation at the Head and Neck outpatient clinic, as well as during the anesthesiological screening before panendoscopy or surgical resection. Patients with a second primary tumor in the head and neck region, tumors outside the upper aerodigestive tract, a cervical metastasis of unknown origin, or a histopathologic diagnosis other than squamous cell carcinoma were excluded.

The Medical Ethics Review Committee of the MUMC (2018-0567) has approved this study and the principles outlined in the Declaration of Helsinki were followed. All data and tissues were handled according to General Data Protection Regulation.

### Tissue Microarrays

FFPE blocks of either the diagnostic biopsy or tumor resection were retrieved and hematoxylin and eosin sections were digitally evaluated with a senior head and neck pathologist (SW or MH), using Pannoramic viewer (3DHISTEC, Budapest, Hungary). Per patient, three 0.6mm tumor tissue cores and one normal epithelium core were selected, placed in a tissue microarray (TMAs), and cut into 5 μm sections.

### RNA *In Situ* Hybridization

**Figure 1:**
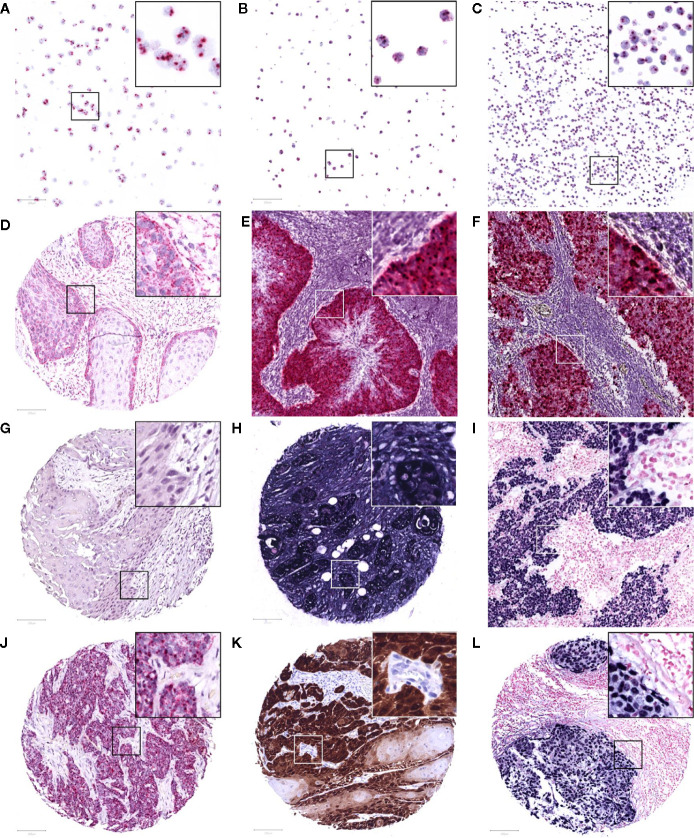
Representative images of RNAscope *in situ* hybridization on cell lines Caski **(A)**, HeLa **(B)**, and WaGa **(C)**, positive for HPV-16, HPV-18, and MCPyV, respectively. A positive and negative control with housekeeping gene transcript PPIB **(D)** and bacterial transcript dapB **(G)** on the TMA, and a control patient positive for HPV-16 and MCPyV **(E, F)**. Positive internal control for housekeeping gene GAPDH **(H)** and a control case of EBV-positive infectious mononucleosis **(I)** following Epstein-Barr virus encoded RNA *in situ* hybridization. Study TMA cores of patients positive for HPV-16 mRNA (at least 1 red punctate dot per tumor cell), p16 immunohistochemistry (>70% strong brown staining of tumor nuclei and cytoplasm), and EBV mRNA (>50% strong blue staining of tumor nuclei) are presented in **(J–L)**, respectively. The images were taken at 200x magnification, an area of 100 μm^2^ is marked in each image and 3x magnified in its top right corner.

To screen for the presence of HPV and MCPyV mRNA, the RNAscope 2.5 RED assay kit and HPV-16/18 or V-MCPyV-LT-ST-Ag probe cocktails (Advanced Cell Diagnostics, Newark, California) were used according to the manufacturer’s instructions. In short, TMA sections were deparaffinized and pretreated with RNAscope Hydrogen Peroxide for 10 min. Antigen retrieval comprised of boiling the slide sections in the provided Target Retrieval Reagents solution at 100°C for 15 min. After washing, the TMAs were dried over night at room temperature and treated for 30 min with RNAscope Protease Plus. In situ hybridization was performed applying four droplets of the provided probes prior to each of the six amplification steps (30 min at 40°C, 15 min at 40°C, 30 min at 40°C, 15 min at 40°C, 30 min at room temperature, and 15 min at room temperature, respectively). After each hybridization step, the slides were washed in the RNAscope wash buffer for 2 min at room temperature. Subsequent to alkaline phosphatase Fast Red chromogenic visualization of hybridized probes, the slides were counterstained with hematoxylin and assessed under a bright field microscope at 200x magnification. Tissue with at least 1 red punctate signal dot in the cytoplasm and/or nucleus of malignant cells was considered to be positive, as suggested by the manufacturer for genes with an expression level varying between 1 to >10 copies per cell. Probes for housekeeping gene transcript human peptidylprolyl isomerase B (Hs-PPIB) and bacterial dihydrodipicolinate reductase gene (dapB) transcript were used as positive and negative controls, respectively. As virus specific positive controls, virus positive tumor tissue was used, in addition to the HPV-18 positive cell line HeLa, HPV-16 positive cell lines SiHa and Caski, and MCPyV positive cell lines MKL-1, MKL-2, and WaGa (**Figure 1**). Negative controls were tumor tissue of a virus negative patient, cell lines MKL-1 and MKL-2 for HPV, and cell lines MCC13 and MCC26 for MCPyV (27).

EBER-ISH was performed using the Dako fluorescein-labeled EBV peptide nucleic acid (PNA) probe mixture and PNA ISH Detection kit (Agilent Technologies, Santa Clara, California) according to the manufacturer’s instructions. Briefly, following TMA section deparaffinization, target retrieval was performed with the Dako Omnis ISH Pre-Treatment solution for 5 min. Subsequently, enzyme pre-treatment was carried out using two steps of 3 min ethanol 96% application and one step of Dako ISH Pepsin for 15 min. Once the provided EBER RNA CISH probe was applied, denaturation at 66°C for 10 min, and hybridization at 45°C for 90 min followed. The slides were washed with the ISH Stringent Wash Buffer for 3 min, and the provided reagents were applied for staining: CISH Endogenous Enzyme Block for 3 min, Anti-FITc-AP for 30 min, and BCIP-NBT Substrate for 15 min. Sections were counterstained with Nuclear Fast Red and analyzed under a bright field microscope at 200x magnification. Strong blue staining of more than 50% of tumor nuclei was considered to be positive. In parallel, a probe for housekeeping gene glyceraldehyde-3-phosphate dehydrogenase (GAPDH) was used to ensure the presence of mRNA in the TMA and a case of EBV-positive infectious mononucleosis served as a positive control (**Figure 1**).

### Immunohistochemistry

Three-μm FFPE TMA sections were subjected to IHC, using primary monoclonal antibodies directed against p16, p53, pRb, and the LTAg of MCPyV (**Table 1**). Immunostainings were performed on a Dako Omnis autostainer (Agilent Technologies) using the EnVision FLEX+ Mouse (LINKER) kit. In short, antigen retrieval was performed on the TMA with sodium citrate-solution (pH 6.0) for p16, or a high pH-buffer (pH 9.0) for p53, pRb, and MCPyV. Endogenous peroxidase was blocked with Dako REAL Peroxidase-Blocking Solution prior to 20 minutes of incubation with the primary antibody. Binding of the antibodies was visualized by an enzymatic reaction with horseradish peroxidase and 3,3’-Diaminobenzidine as substrate, producing a brown precipitate (**Figure 1**). Slides were counterstained with hematoxylin and evaluated under a bright field microscope by two independent assessors, blinded for patients’ clinical characteristics. In case of dissonance between the assessors, a third assessor evaluated the staining and agreement was reached by discussion. For p16, strong homogenous staining in the cytoplasm and nuclei of >70% of the tumor cells was considered to be overexpression (28). p53 staining was assessed as 0-mutant type (0% nuclear staining), mutant-type overexpression (>70% strong nuclear staining in the non-keratinizing tumor cells), or wild-type (heterogeneous nuclear staining) (29). For pRb, nuclear staining in <25% of tumors cells was evaluated as loss of pRb (30). MCPyV LTAg was considered positive if >10% of nuclei were stained (31). For p16, a case of HPV-positive OPSCC was used as a control, and for p53 and pRb normal tonsil tissue was used. MCPyV positive cell lines MKL-1, MKL-2, and WaGa served as positive controls for the LTAg of MCPyV.

**Table 1:** Immunohistochemistry primary antibodies and evaluation criteria.

Antibody characteristics		Source	Clone	Dilution	Retrieval	Localization	Evaluation criteria	
Antibody	Company						Cut off	References
p16	Immunologic	Monoclonal, Mouse	MX007	1:200	Citrate (pH 6.0)	Nuclear and cytoplasmic	>70%	(28)
p53	Dako Omnis	Monoclonal, Mouse	DO-7	Ready-to-use	High pH buffer (pH 9.0)	Nuclear	0% >70%	(29)
pRb	Leica Biosystems	Monoclonal, Mouse	13a10	1:100	High pH buffer (pH 9.0)	Nuclear	≥25%	(30)
Large T-antigen	Santa Cruz Biotechnology	Monoclonal, Mouse	CM2B4	1:50	High pH buffer (pH 9.0)	Nuclear	>10%	(31)

### Human Papillomavirus-Specific Polymerase Chain Reaction

Of patients with a positive result for HPV RNA-ISH and/or p16 IHC, DNA was isolated from eight 5-μm FFPE whole tissue sections with the Maxwell RSC DNA FFPE kit (Promega, Madison, Wisconsin). DNA concentrations were determined using the Quantus Fluorometer and the QuantiFluor ONE dsDNA system (Promega). Next, 250 ng DNA was added to 1 ml SurePath preservative fluid (VWR International, Amsterdam, Netherlands) and used for HPV-DNA analysis utilizing the COBAS 4800 platform (Roche, Basel, Switzerland), according to the manufacturer’s instructions. The COBAS 4800 tests specifically for HPV-16, HPV-18, and a combination of 12 other HR-HPV types (31, 33, 35, 39, 45, 51, 52, 56, 58, 59, 66, and 68). Results were considered reliable when the controls were labeled “valid”. Housekeeping gene β-globin was used as a control for the human DNA, in addition to DNA samples of an HPV-positive and HPV-negative tumor.

### Statistical Analysis

Patients were considered to be HPV or MCPyV positive when the virus was detected with at least two techniques (ISH, IHC and/or PCR). EBV presence was based on the EBER-ISH result. Differences in clinical parameters between virus positive and virus negative patients were evaluated by Mann-Whitney U test for age because of a non-normal distribution, Fisher’s exact test for binominal variables (sex, M-stage, recurrence, p16, pRb), and the Fisher-Freeman-Halton exact test for tumor location, T-stage, N-stage, and p53 because of expected counts of less than five. The 5-year DFS and OS were estimated with Kaplan-Meier curves and differences between virus positive and negative tumors were determined by log rank test. DFS was defined as the last date of treatment until the biopsy date of a histologically proven recurrence or second primary tumor in the head and neck region. OS was defined as the time between the primary tumor biopsy date and death. Censoring took place when patients were lost to follow-up, deceased without recurrent disease for DFS, or at the cut-off point of 60 months. All clinical and pathological parameters were assessed in bivariate analysis regarding survival. Variables with significant (*p* < 0.05) or near significant (*p* < 0.1) relationships were evaluated in multinomial logistic regression to assess predictors for DFS and OS. Analyses were performed using IBM SPSS Statistics 25.0 (IMB corp., Armonk, NY) and a *p*-value of <0.05 was considered to be statistically significant.

## Results

A total of 119 patients were included in this study. These patients had a median age of 74.9 years (inter quartile range = 14.6 years) and were mainly women (78%) with a tumor of the oral cavity (80%) and no regional or distant metastases (66 and 95%, respectively). Thirty-one patients (26%) had recurrent disease within 5 years (**Table 2**).

**Table 2:** Comparison of clinical characteristics between human papillomavirus (HPV) and Epstein–Barr virus (EBV) positive and negative head and neck squamous cell carcinoma in non-smokers and non-drinkers.

Clinical characteristics		Total (n = 119)		HPV-positive (n = 12)		HPV-negative (n = 107)		p-value	EBV-positive (n = 3)		EBV-negative (n = 116)		p-value
Age (years)	Median (interquartile range)	74.9	(14.6)	67.3	(13.2)	76.2	(14.7)	0.003	48.0	(NA*)	75.3	(14.1)	0.062
		*n*	*(%)*	*n*	*(%)*	*n*	*(%)*		*n*	*(%)*	*n*	*(%)*	
Sex	Female	93	(78)	10	(83)	83	(78)	1.0	2	(67)	91	(78)	0.53
	Male	26	(22)	2	(17)	24	(22)		1	(33)	25	(22)	
Location	Hypopharynx	1	(0.8)	0	(0)	1	(0.9)	<0.001	0	(0)	1	(0.9)	<0.001
	Larynx	9	(7.6)	0	(0)	9	(8.4)		0	(0)	9	(7.8)	
	Nasopharynx	4	(3.4)	1	(8.3)	3	(2.8)		3	(100)	1	(0.9)	
	Oral cavity	95	(80)	1	(8.3)	94	(88)		0	(0)	95	(82)	
	Oropharynx	10	(8.4)	10	(83)	0	(0)		0	(0)	10	(8.6)	
T-stage	1	33	(28)	3	(25)	30	(28)	0.35	1	(33)	32	(28)	1.0
	2	36	(30)	2	(17)	34	(32)		1	(33)	35	(30)	
	3	13	(11)	3	(25)	10	(9.3)		0	(0)	13	(11)	
	4	37	(31)	4	(33)	33	(31)		1	(33)	1	(31)	
N-stage	0	78	(66)	5	(42)	73	(68)	0.004	0	(0)	78	(67)	0.061
	1	18	(15)	0	(0)	18	(17)		1	(33)	17	(15)	
	2	21	(18)	7	(58)	14	(13)		2	(67)	19	(16)	
	3	2	(1.7)	0	(0)	2	(1.9)		0	(0)	2	(1.7)	
M-stage	0	113	(95)	11	(92)	102	(95)	0.42	3	(100)	111	(96)	1.0
	1	6	(5.0)	1	(8.3)	5	(4.7)		0	(0)	5	(4.3)	
Recurrence	Yes	31	(26)	2	(17)	29	(27)	0.73	2	(67)	29	(25)	0.17
	No	88	(74)	10	(83)	78	(73)		1	(33)	87	(75)	

*Not applicable because of small number of patients.

### Human Papillomavirus

ISH on the TMAs showed HPV-16/18 mRNA expression in tumors of ten patients. All of these tumors showed p16 overexpression by IHC as well, in addition to five tumors with no HPV-16/18 mRNA expression. COBAS analysis on HR-HPV DNA in these 15 patients detected the presence of HPV-16 in ten tumors and another HR-HPV type in one other case. For patient 61, the quality of the DNA was insufficient for COBAS analysis. This resulted in a total of 12 tumors being HPV-positive based on at least two detection techniques (**Table 3**).

**Table 3:** Demographics and viral analysis results of 15 virus positive tumors in non-smokers and non-drinkers.

Virus	Study ID	Age (years)	Sex	Tumor location	T	N	M	Recurrence	ISH	p16 IHC	COBAS PCR
HPV	1	56.4	Male	Oropharynx	3	2	0	No	+^*^	+	HPV-16
	3	53.9	Female	Oropharynx	1	2	0	No	+^*^	+	HPV-16
	4	71.5	Male	Oropharynx	4	2	0	No	+^*^	+	HPV-16
	5	76.6	Female	Oropharynx	2	2	0	No	+^*^	+	HPV-16
	9	69.5	Female	Oropharynx	3	0	0	No	-^*^	+	HR-HPV
	16	68.3	Female	Oropharynx	1	0	0	No	+^*^	+	HPV-16
	21	71.8	Female	Oropharynx	4	2	1	Yes	+^*^	+	HPV-16
	29	57.8	Female	Nasopharynx	2	0	0	No	+^*^	+	HPV-16
	32	63.5	Female	Oral cavity	4	0	0	No	-^*^	+	HPV-16
	40	67.9	Female	Oropharynx	4	2	0	No	+^*^	+	HPV-16
	61	66.6	Female	Oropharynx	3	0	0	Yes	+^*^	+	Invalid
	122	57.9	Female	Oropharynx	1	2	0	No	+^*^	+	HPV-16
EBV	6	48.0	Male	Nasopharynx	4	2	0	Yes	+^†^	NA	NA
	13	47.3	Female	Nasopharynx	2	1	0	No	+^†^	NA	NA
	18	74.6	Female	Nasopharynx	1	2	0	Yes	+^†^	NA	NA

ISH, in situ hybridization; IHC, immunohistochemistry; PCR, polymerase chain reaction; +, positive; -, negative; ^*^, using RNAscope; ^†^, using EBER-ISH; HR-HPV, high-risk human papilloma virus; EBV, Epstein-Barr virus; NA, not applicable.

Compared to HPV-negative tumors, HPV-positive tumors were associated with lower age (67.3 versus 76.2 years old, *p* = 0.003), oropharyngeal origin (83% versus 0%, *p* < 0.001), and N2-stage (58% versus 13%, *p* = 0.004) (**Table 2**). All oropharyngeal tumors (n = 10) were HPV-positive, in addition to one OSCC of the alveolar process and a NPSCC. Although the OSCC case showed HPV-16 DNA and p16 overexpression, no mRNA was detected with ISH, neither on the TMA nor on a whole section.

**Figure 2:**
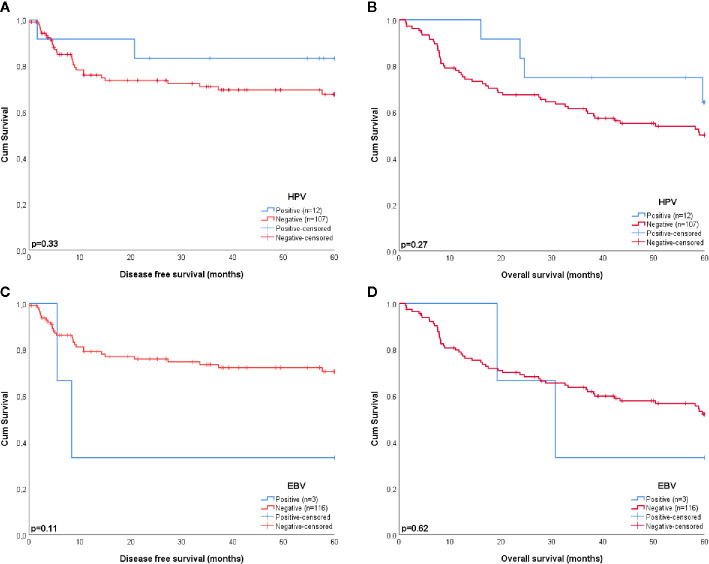
Kaplan-Meier curves estimating the survival of patients with HPV **(A, B)** and EBV **(C, D)** positive and negative tumors. HPV and EBV were no significant predictors for a better disease free (*p* = 0.33 and *p* = 0.11, respectively) or overall survival (*p* = 0.27 and *p* = 0.62, respectively).

Four of the twelve patients with HPV-positive tumors (33%) died within five years, two of which had recurrent disease. For patient 1 there were no details recorded on the cause of death (OS = 59.6 months), patient 5 died of the complications of an aortic valve prosthesis endocarditis (OS = 23.7 months), patient 21 received palliative treatment because of distant metastasis (DFS = 1.6 months), and patient 61 developed liver metastases 20.1 months after initial therapy (**Table 3**). The presence of HPV was no predictor for a better DFS or OS (*p* = 0.33 and *p* = 0.27, respectively), compared to HPV-negative HNSCC in NSND (**Figures 2A, B**). A younger age at cancer diagnosis (*p =* 0.008), T1 stage (*p =* 0.0047), and N0 or N1 stage (both *p <* 0.001) were retained in the best multivariable model as predictors for OS in HNSCC of NSND (**Supplementary table 1**, **Table 4**). The model explained 35% of the variation (model fit: omnibus test of model coefficients: *X^2^* = 36.0 and *p <* 0.001; M-stage was omitted because of low case numbers).

**Table 4:** Multivariable analysis of predictors for 5-year overall survival in non-smokers and non-drinkers with head and neck squamous cell carcinoma.

Parameter	Coefficient (β)	Standard error	Wald X^2^	OR	95% CI		p-value
					Lower	Upper	
Age	0.053	0.020	7.0	1.05	1.0	1.1	0.008
*T-stage (reference T4)*							
T1	-1.2	0.86	3.9	0.31	0.099	0.99	0.047
T2	-0.67	0.54	1.5	0.51	0.18	1.5	0.22
T3	0.79	0.74	1.1	2.2	0.52	9.4	0.29
*N-stage (reference N3)*							
N0	-19	0.59	1081	<0.001	<0.001	<0.001	<0.001
N1	-17	0.78	506	<0.001	<0.001	<0.001	<0.001
N2	-18	0.00	NA	<0.001	<0.001	<0.001	NA

NA, not available because of small number of cases.

Nagelkerke R^2^ = 0.35.

HPV-positive tumors showed significantly more often p16 overexpression, p53 wild-type expression, and loss of pRb than HPV-negative tumors (p16: 100 versus 2.8%, *p* < 0.001; p53: 83 versus 27%, *p* < 0.001; pRb: 83 versus 19%, *p* < 0.001) (**Table 5**).

**Table 5:** Expression of cell cycle proteins p16, p53, and pRb in virus positive and negative tumors.

Cell cycle protein		Virus positive		Virus negative		p-value	HPV-positive		HPV-negative		p-value	EBV-positive		EBV-negative		p-value
		n	(%)	n	(%)		n	(%)	n	(%)		n	(%)	n	(%)	
p16	Overexpression	12	(80)	3	(2.9)	<0.001	12	(100)	3	(2.8)	<0.001	0	(0)	15	(13)	1.0
	No overexpression	3	(20)	101	(97)		0	(0)	104	(97)		3	(100)	101	(87)	
p53	Mutant-type overexpression	2	(13)	51	(49)	0.002	0	(0)	53	(50)	<0.001	2	(67)	51	(44)	0.80
	Wild-type	11	(73)	28	(27)		10	(83)	29	(27)		1	(33)	38	(33)	
	0-mutant type	2	(13)	25	(24)		2	(17)	25	(23)		0	(0)	27	(23)	
pRb	Positive (preserved expression)	5	(33)	84	(81)	<0.001	2	(17)	87	(81)	<0.001	3	(100)	86	(74)	0.57
	Loss	10	(67)	20	(19)		10	(83)	20	(19)		0	(0)	30	(26)	

HPV, human papillomavirus; EBV, Epstein–Barr virus.

### Epstein–Barr Virus

Three tumors were EBV positive as detected by EBER-ISH. These patients all had a tumor of the nasopharynx (100 versus 0.9% in EBV-negative tumors, *p* < 0.001), resulting in virus positivity of all four nasopharyngeal tumors in this cohort (three containing EBV and one HPV). Two of the three patients (67%) with EBV-positive tumors were below 50 years of age and non-Caucasian (Northern African and East Asian) and they all had regional metastases (**Tables 2**, **3**).

Patient 6 and 18 were both diagnosed with recurrent disease, the former with distant metastases 8.3 months after chemoradiotherapy and the latter with regional metastases 5.5 months after locoregional radiotherapy, which eventually led to their death. Although this resulted in a 33% 5-year survival for patients with EBV-positive tumors, EBV was no significant predictor for DFS or OS in this cohort, compared to EBV-negative HNSCC (*p* = 0.11 and *p* = 0.62, respectively) (**Figures 2C, D**).

None of the EBV-positive tumors showed p16 overexpression, all were positive for pRb and two of the three tumors (66%) showed p53 mutant-type overexpression. This did not differ significantly from cell cycle protein expression in EBV-negative tumors, most probably because of the small number of EBV-positive tumors (**Table 5**).

### Merkel Cell Polyomavirus

MCPyV was not detected in any of the samples with RNA-ISH or IHC against the LTAg of MCPyV.

### Virus Negative Tumors

None of the squamous cell carcinomas of the oral tongue (OTSCC) (n = 39), larynx (LSCC) (n = 9), or hypopharynx (HPSCC) (n = 1) showed involvement of HPV, EBV, or MCPyV. Except for one tumor of the alveolar process, all other OSCC (n = 54) were virus negative as well (**Table 2**). Although the patients with virus negative tumors were relatively old (mean age of 75 years) when being diagnosed with HNSCC, the 5-year OS of these patients was still 50% (**Figure 2**).

The three patients with virus negative OSCC containing p16 overexpression all had recurrent disease within 6 months after surgical resection [a T4N1M0 floor of mouth tumor, after an irradical resection the patient wished no further treatment (DFS = 0 months), OS = 27.4 months; a T4N0M0 oral cavity tumor (not otherwise specified), DFS = 5.4 months after resection, OS = 8.2 months; a T2N1M0 retromolar triangle tumor, DFS 4.8 months after resection, OS = 11.8 months]. Two of these tumors showed loss of pRb expression. A poor DFS (2.3, 8.8, and 20.3 months) was also found in three other tumors in this cohort with some p16 expression (>50%): all OTSCC with a pRb expression above 50%. Apart from one LSCC, all tumors with loss of pRb were OSCC (19/20) without any expression of p16. A younger age at cancer diagnosis (*p =* 0.021) and N0 or N1 stage (both *p <* 0.001) were retained in the best multivariable model as predictors for OS in NSND with virus negative OSCC (**Supplementary Table 2**, **Table 6**). The model explained 31% of the variation (model fit: omnibus test of model coefficients: *X^2^* = 24.4 and *p <* 0.001; M-stage and p16 were omitted because of low case numbers).

**Table 6:** Multivariable analysis of predictors for 5-year overall survival in non-smokers and non-drinkers with virus negative oral squamous cell carcinoma.

Parameter	Coefficient (β)	Standard error	Wald X^2^	OR	95% CI		p-value
					Lower	Upper	
Age	0.051	0.022	5.3	1.05	1.0	1.1	0.021
*N-stage (reference N3)*							
N0	-20	0.72	803	<0.001	<0.001	<0.001	<0.001
N1	-18	0.89	414	<0.001	<0.001	<0.001	<0.001
N2	-19	0.00	NA	<0.001	<0.001	<0.001	NA

NA, not available because of small number of cases.

Nagelkerke R^2^ = 0.31.

Over 70% (76/104) of virus negative tumors showed mutant-type p53 expression, with 0-type mutant expression in 20% (19/94) of OSCC, 56% (5/9) of LSCC, and 100% (1/1) of HPSCC, and mutant-type overexpression in 52% (49/94) of OSCC and 22% (2/9) of LSCC (**Table 5**). Cell cycle protein expression was no predictor for a better DFS or OS in patients with virus negative tumors (data not displayed).

## Discussion

The objective of this study was to determine if HPV, EBV, and MCPyV play a role in head and neck carcinogenesis of NSND, the role of cell cycle proteins p16, p53, and pRb regarding viral presence, and the influence of these viruses and proteins on patient survival. In this cohort of 119 NSND, the ten oropharyngeal (100%) and four nasopharyngeal (100%) tumors contained either HPV or EBV. Besides one oral cavity tumor, all other specimens of the oral cavity, hypopharynx, and larynx were HPV, EBV, and MCPyV-negative. Virus positivity did not predict better disease free or overall survival. Regarding cell cycle protein expression, HPV-positive tumors showed more p16 overexpression, wild-type p53 expression, and loss of pRb compared to HPV-negative tumors. OSCC with >70% p16 expression had a poor DFS and OS, with loss of pRb in two of the three cases. The other pRb negative tumors were mainly OSCC as well and did not show p16 expression. Mutant type p53 expression was observed in over 70% of virus negative HNSCC.

As the worldwide HPV prevalence is rising, a wide range has been reported in the literature, ranging from 20% in OPSCC patients from Eastern Asia or Central America, to over 50% in Europe and Australia, based on HPV DNA combined with E6*I mRNA or p16 IHC (5, 6). A recent systematic review specifically analyzing patients without tobacco or alcohol consumption reported an OPSCC HPV prevalence of over 60% in non-smokers and over 40% in non-drinkers, compared to 20% in smokers and drinkers, based on at least two detection techniques (combining PCR, ISH, IHC, or sequencing) (25). This is a lower prevalence than the 100% (10/10) HPV infections of OPSCC in the current study. Possibly, the low number of OPSCC in the current study explains the 100% prevalence, as it could be a coincidence that they were all HPV-positive. Nonetheless, it is acknowledged that HPV plays an increasingly substantial role in OPSCC carcinogenesis of patients without the traditional risk factors. The HPV prevalence of 1.8% (2/109) in non-OPSCC as found in the current study is comparable to the low prevalence in other studies (32–35).

In this study, 4.2% (4/95) of OSCC showed p16 overexpression with IHC, and one of those contained HPV-16 DNA following COBAS analysis resulting in HPV-positivity according to detection by two methods. p16 overexpression could result from loss of pRb function *via* structural alterations, or maybe as a result of other oncogenic viruses affecting pRb expression that we are not aware of (36). Lechner and colleagues speculated that high levels of protein p16 could also occur in cells irrespective of pRb expression or HPV-positivity, as a result of enrichment for *NSD1* mutations in *CDKN2A* wild-type tumors (36, 37). *NSD1* is coding for Histone H3K36 methyltransferase, which is associated with DNA hypomethylation, resulting in p16 overexpression when mutated by not being able to regulate its expression *via* methylation anymore (36). Indeed, p16 overexpression has been reported in non-OPSCC, without a correlation to HPV infection nor as a predictor for survival (34, 38). Therefore, The College of American Pathologists does not endorse routine p16 screening for non-OPSCC (39). As the one HPV-positive OSCC in this cohort had no loss of pRb and lacked HPV mRNA in a whole section following RNAscope ISH analysis, the p16 overexpression could be a result of *CDKN2A* mutation, and HPV a commensal with the HPV DNA not located in the tumor cells but in the adjacent mucosal epithelial cells (40). The three HPV-negative OSCC with p16 overexpression in the current study had a poor DFS. This could be a result of pRb loss, although for the whole study group p16 overexpression was no significant predictor for survival. Additionally, HNSCC could be the result of a genetic predisposition. OSCC has been associated with specific *CDKN2A* germline mutations, accompanied with loss of heterogeneity of the wild-type allele, in a small fraction of young NSND (41).

The 5-year OS of HNSCC patients in general is 40–50%, whereas it is 70–80% for patients with HPV-positive OPSCC (2, 42). In this study, there was no significant difference in OS or DFS between virus positive and virus negative patients. However, it is not certain if the survival comparison of HPV-positive versus HPV-negative HNSCC was one of HPV-positivity or of tumor location (OPSCC versus non-OPSCC), as in this cohort HPV-negative tumors were exclusively non-OPSCC. The same applies to EBV-positive tumors, which were all NPSCC. Therefore, these survival analyses should be interpreted with caution. The 5-year OS of 67% for patients with HPV-positive tumors was lower than expected, but these patients were relatively old with a median age of 67.3 years. Patients with HPV-negative HNSCC (53%) did not differ significantly in 5-year OS from patients with HPV-positive OPSCC, even though they had a median age of almost 9 years older than the patients with HPV-positive tumors (76.2 versus 67.3 years). So, considering their age at the time of HNSCC diagnosis, the HPV-negative NSND, mainly with OSCC, had a relatively good 5-year OS. Nevertheless, a young age at the time of cancer diagnosis was predictive of a better OS in both the virus negative OSCC group and the whole NSND cohort, besides a T1 stage and a N0 or N1 stage.

Viral association in all four NPSCC patients of this cohort was as expected. Although the worldwide incidence of EBV related NPSCC has been decreasing over the past decade, the prevalence is still high with almost 100% in endemic regions (southern China, Southeast Asia, Northern Africa, and the Mediterranean basin), and 60–85% in non-endemic regions (9, 43–46). NPSCC infections with HPV are less common, and have been reported in studies from non-endemic regions like West Africa and Europe (outside the Mediterranean basin), with a prevalence between 1.6–16% (43, 45, 47, 48). Based on 517 U.S. NPSCC patients with known HPV testing (34.8% HPV-positive) in the Surveillance, Epidemiology and End Results database, predictors for HPV-positivity in NPSCC have been established: being younger than 25-years-old, Caucasian (rather than East-Asian or other ethnicities), an AJCC-7 stage other than stage 1, and no distant metastases (M0) (49). Indeed, the one patient in our cohort with HPV-positive NPSCC was a Caucasian patient with stage 2 disease.

Expression of cell cycle proteins p16 and pRb was as expected in the HPV-positive OPSCC of this cohort, with overexpression of the former and loss of the latter. Although p53 is usually degraded by HPV’s viral oncoprotein E6, only two HPV-positive tumors showed 0-mutant type p53 expression, whereas the other 10 showed wild-type p53 expression. This is in agreement with earlier observations, where there was p53 expression in 7/10 HPV-positive tumors of non-smokers, despite absence of *TP53* mutations (50). Possible explanations for this finding are virally induced processes, such as hypoxia, oxidative stress, or impaired repair of double strand DNA breaks (50, 51). On the other hand, up to 55–75% of HPV-negative OSCC contain *TP53* mutations, which is comparable to the 72% of HPV-negative OSCC showing mutant-type p53 expression (either 0-type or overexpression) in the current study (36, 52). The EBV-positive tumors were all without p16 overexpression and with preserved pRb expression. Zhang and colleagues reported that the cell cycle pathway is the most deregulated pathway in NPSCC in comparison to non-tumor nasopharyngeal epithelium, with down-regulation of p16 and up-regulation of pRb (19). Although the precise mechanism of p16 inactivation by EBV in NPSCC remains unclear, it has been suggested that the Late Membrane Protein 1 of EBV could inhibit p16 expression and induce pRb phosphorylation, promoting the progression of the G1/S phase (19). This is in accordance with the cell cycle protein expressions found in the current study.

No MCPyV was detected in the current study, which contrasts earlier findings. Hamiter and colleagues performed PCR using specific primers for the regulatory and LTAg of MCPyV, followed by DNA sequence analysis to confirm viral presence in 6/21 (29%, three were NSND) patients with OTSCC (16). Although the OTSCC group in the current study was almost twice the size (n = 39), no MCPyV presence was detected. Other studies report MCPyV DNA in 4–50% of HNSCC based on quantitative real-time PCR, though with low viral loads (14, 15, 17). This discrepancy could be the result of differences in sensitivity between the used detection methods (RNA-ISH and LTAg IHC versus DNA PCR and sequencing). However, the MCPyV presence in the literature was solely based on PCR and was not confirmed with another detection method. Nevertheless, the significance of MCPyV presence in HNSCC has yet to be determined, but our data strengthen the premise that MCPyV is not likely to play an important role in head and neck carcinogenesis.

HPV has its clinical relevance in routine practice as a prognostic marker in OPSCC, with a better DFS and OS, in addition to an improved radiosensitivity compared to HPV-negative OPSCC because of altered DNA repair, reduced hypoxic regions, and an increased cellular immune response (3, 53). With the conduction of multiple phase III de-escalation trials for HPV-positive OPSCC, and the high HPV prevalence in OPSCC of NSND, the treatment of NSND may be affected in the near future (4). For EBV-positive NPSCC, treatment usually consists of radiotherapy, with or without chemotherapy. Besides the anti-tumor effects of radiotherapy based on direct and indirect DNA damage, it also induces an immune response comprising of a network of immune-stimulatory and –inhibitory signals like up-regulation of immune checkpoint proteins such as PD-1/PD-L1 (54). Consequently, there are a number of clinical trials evaluating the incorporation of immune checkpoint inhibitors in the treatment of EBV-positive NPSCC (10, 54). Mutant p53 has been associated with resistance to chemoradiation in OSCC and an increased risk of locoregional recurrence and metastases (55). Since NSND mainly have OSCC and frequent mutant-type p53 expression (as presented in this study), p53 could be used to predict therapy failure in case of recurrent disease.

One of the limitations of this study was that the definition of when a patient was a non-smoker and non-drinker was collected retrospectively from their medical records. Nevertheless, a strict definition was used (for example with exclusion of patients with “sporadic” alcohol consumption), based on a standard history taking template including specific questions on any current or previous tobacco and alcohol consumption, during two separate hospital visits (Head and Neck outpatient clinic and Anesthesiology screening). Secondly, there was a small group of patients with tumors at anatomical sites other than the oral cavity (OSCC). In combination with the strict NSND definition, this resulted in no virus negative OPSCC and NPSCC. However, it has been reported that NSND are mainly patients with OSCC, so a small number of tumors outside the oral cavity was expected (25, 56). Thirdly, there might be a higher percentage of HNSCC positive for HPV DNA in this cohort, because COBAS PCR analysis was not performed on all samples. Conversely, HPV is only considered to be predictive for survival when being transcriptionally active, and since all tumors were tested on HPV-16/18 mRNA and p16 IHC, we expect to have detected the tumors with biologically active HPV infection (7, 8). Finally, some of the FFPE material was rather old (>25 years of storage), which is known to often result in breakdown of the nucleic acids. Indeed, DNA quality of DNA extracted from one tumor was insufficient for COBAS analysis. Nevertheless, the TMA blocks were freshly sectioned before ISH, IHC, or PCR, and all the positive controls were adequate.

## Conclusion

A high prevalence of HPV and EBV was observed in OPSCC and NPSCC of NSND respectively, but not in HNSCC outside these locations. Although a significant role of oncogenic viruses would be assumed in this specific patient group lacking the traditional risk factors for developing HNSCC, HPV, EBV, and MCPyV were not detected in this relatively large cohort of 95 OSCC apart from one case, using clinically relevant cut-off values. This argues against a central role of these viruses in the etiopathogenesis of oral, hypopharyngeal, and laryngeal squamous cell carcinoma in this specific patient group. With ongoing de-escalation trials for HPV-positive OPSCC and trials for immune checkpoint inhibitors in the treatment of EBV-positive NPSCC, the treatment of NSND with tumors at those locations may change in the near future. However, for the majority of NSND with virus negative OSCC, more research is needed to understand the carcinogenic mechanisms in order to consider targeted therapeutic options.

## Data Availability Statement

The raw data supporting the conclusions of this article will be made available by the authors, without undue reservation.

## Ethics Statement

The studies involving human participants were reviewed and approved by Maastricht University Medical Center (METc 2018-0567). Written informed consent for participation was not required for this study in accordance with the national legislation and the institutional requirements.

## Author Contributions

All authors have contributed substantially to the conception and design (FM, SW, BK, and ES), acquisition of data (FM, FK, FJ, and FF), analysis and interpretation of data (FM, SW, RB, AH, MH, BK, and ES); drafting the article (FM, FJ, and ES) or revising it critically for important intellectual content (FK, FF, SW, RB, AH, MH, and BK); final approval of the version to be published (FM, FK, FJ, FF, SW, RB, AH, MH, BK, and ES); and agree to be accountable for all aspects of the work in ensuring that questions related to the accuracy or integrity of any part of the work are appropriately investigated and resolved. All authors contributed to the article and approved the submitted version.

## Conflict of Interest

SW receives a research grant from Roche, MSD, AstraZeneca, Pfizer, Nextcure, Amgen, and Bayer. BK and ES receive a research grant from Pfizer and Novartis. The funders had no role in the design of the study, in the collection, analyses, or interpretation of the data, in the writing of the manuscript, or in the decision to publish the results.

The remaining authors declare that the research was conducted in the absence of any commercial or financial relationships that could be construed as a potential conflict of interest.
