# Supplementary material for: Presence of Human Papillomavirus and Epstein–Barr Virus, but Absence of Merkel Cell Polyomavirus, in Head and Neck Cancer of Non-Smokers and Non-Drinkers
Source: Front Oncol. 2021 Jan 20;10:560434. doi: 10.3389/fonc.2020.560434 (PMC7855709; doi:10.3389/fonc.2020.560434)
Supplement: Supplementary file 1 [file Table_1.docx]

| **Supplementary table 1.** Predictors for 5-year DFS and OS in non-smokers and non-drinkers with HNSCC | | | | | | | | | | | | | |
| --- | --- | --- | --- | --- | --- | --- | --- | --- | --- | --- | --- | --- | --- |
| *Clinical characteristics* | | *Total (n=119)* | | *5-year DFS (n=88)* | | *Recurrence (n=31)* | | *p-value* | *5-year OS (n=65)* | | *Death (n=54)* | | *p-value* |
| Age (years) | Median (IQR) | 74.9 | (14.6) | 75.5 | (16.0) | 74.2 | (12.9) | 0.51 | 70.8 | (18.2) | 77.4 | (11.2) | 0.007 |
|  |  | *n* | *(%)* | *n* | *(%)* | *n* | *(%)* |  | *n* | *(%)* | *n* | *(%)* |  |
| Sex | Female | 93 | (78) | 70 | (80) | 23 | (74) | 0.61 | 12 | (19) | 14 | (26) | 0.38 |
|  | Male | 26 | (22) | 18 | (21) | 8 | (26) |  | 53 | (82) | 40 | (74) |  |
| Location | Hypopharynx | 1 | (0.8) | 1 | (1.1) | 0 | (0) | 0.72 | 0 | (0) | 1 | (1.9) | 0.53 |
|  | Larynx | 9 | (7.6) | 6 | (6.8) | 3 | (9.7) |  | 3 | (4.6) | 6 | (11) |  |
|  | Nasopharynx | 4 | (3.4) | 2 | (2.3) | 2 | (6.5) |  | 2 | (3.1) | 2 | (3.7) |  |
|  | Oral cavity | 95 | (80) | 71 | (81) | 24 | (77) |  | 54 | (83) | 41 | (76) |  |
|  | Oropharynx | 10 | (8.4) | 8 | (9.1) | 2 | (6.5) |  | 6 | (9.2) | 4 | (7.4) |  |
| T-stage | 1 | 33 | (28) | 25 | (28) | 8 | (26) | 0.76 | 26 | (40) | 7 | (13) | 0.003 |
|  | 2 | 36 | (30) | 27 | (31) | 9 | (29) |  | 20 | (31) | 16 | (30) |  |
|  | 3 | 13 | (11) | 8 | (9.1) | 5 | (16) |  | 4 | (6.2) | 9 | (17) |  |
|  | 4 | 37 | (31) | 28 | (32) | 9 | (29) |  | 15 | (23 | 22 | (41) |  |
| N-stage | 0 | 78 | (66) | 17 | (55) | 61 | (69) | 0.31 | 52 | (80) | 26 | (48) | 0.001 |
|  | 1 | 18 | (15) | 7 | (23) | 11 | (13) |  | 5 | (7.7) | 13 | (24) |  |
|  | 2 | 21 | (18) | 7 | (23) | 14 | (16) |  | 8 | (12) | 13 | (24) |  |
|  | 3 | 2 | (1.7) | 0 | (0) | 2 | (2.3) |  | 0 | (0) | 2 | (3.7) |  |
| M-stage | 0 | 113 | (95) | 87 | (99) | 27 | (87) | 0.016 | 65 | (100) | 49 | (91) | 0.017 |
|  | 1 | 2 | (5.0) | 1 | (1.1) | 4 | (13) |  | 0 | (0) | 5 | (9.3) |  |
| HPV | Positive^*^ | 12 | (10) | 10 | (11) | 2 | (6.5) | 0.73 | 8 | (12) | 4 | (7.4) | 0.54 |
|  | Negative | 107 | (90) | 78 | (89) | 29 | (94) |  | 57 | (88) | 50 | (93) |  |
| EBV | Positive^†^ | 3 | (2.5) | 87 | (99) | 2 | (6.5) | 0.17 | 1 | (1.5) | 2 | (3.7) | 0.59 |
|  | Negative | 116 | (98) | 1 | (1.1) | 29 | (94) |  | 64 | (99) | 52 | (96) |  |
| p16 | No overexpression | 15 | (13) | 10 | (11) | 5 | (16) | 0.53 | 57 | (88) | 47 | (87) | 1.0 |
|  | Overexpression | 104 | (87) | 78 | (89) | 26 | (84) |  | 8 | (12) | 7 | (13) |  |
| p53 | 0-mutant type | 27 | (23) | 19 | (22) | 8 | (26) | 0.80 | 11 | (17) | 16 | (30) | 0.25 |
|  | Wild-type | 39 | (33) | 30 | (34) | 9 | (29) |  | 22 | (34) | 17 | (32) |  |
|  | Mutant-type | 53 | (45) | 39 | (44) | 14 | (45) |  | 32 | (49) | 21 | (39) |  |
| pRb | Loss | 30 | (25) | 21 | (24) | 9 | (29) | 0.63 | 14 | (22) | 16 | (30) | 0.40 |
|  | Positive | 89 | (75) | 67 | (76) | 22 | (71) |  | 51 | (79) | 38 | (70) |  |
| DFS: disease free survival; OS: overall survival; HNSCC: head and neck squamous cell carcinoma; IQR: inter quartile range; ^*^: using at least two detection methods (RNAscope ISH, COBAS PCR, and/or p16 IHC); ^†^; using EBER-ISH | | | | | | | | | | | | | |
|  |  |  |  |  |  |  |  |  |  |  |  |  |  |
| **Supplementary table 2.** Predictors for 5-year DFS and OS in non-smokers and non-drinkers with virus negative OSCC | | | | | | | | | | | | | |
| *Clinical characteristics* | | *Total (n=94)* | | *5-year DFS (n=70)* | | *Recurrence (n=24)* | | *p-value* | *5-year OS (n=53)* | | *Death (n=41)* | | *p-value* |
| Age (years) | Median (IQR) | 76.7 | (14.0) | 77.7 | (13.7) | 73.8 | (13.4) | 0.31 | 77.4 | (11.2) | 70.8 | (18.2) | 0.049 |
|  |  | *n* | *(%)* | *n* | *(%)* | *n* | *(%)* |  | *n* | *(%)* | *n* | *(%)* |  |
| Sex | Female | 16 | (17) | 59 | (84) | 19 | (79) | 0.54 | 9 | (17) | 7 | (17) | 1.0 |
|  | Male | 78 | (83) | 11 | (16) | 5 | (21) |  | 44 | (83) | 34 | (83) |  |
| T-stage | 1 | 28 | (29) | 21 | (30) | 7 | (29) | 0.98 | 22 | (42) | 6 | (15) | 0.025 |
|  | 2 | 32 | (34) | 23 | (33) | 9 | (38) |  | 17 | (32) | 15 | (37) |  |
|  | 3 | 7 | (7.4) | 5 | (7.1) | 2 | (8.3) |  | 3 | (5.7) | 4 | (9.8) |  |
|  | 4 | 27 | (29) | 21 | (30) | 6 | (25) |  | 11 | (21) | 16 | (39) |  |
| N-stage | 0 | 64 | (68) | 51 | (73) | 13 | (54) | 0.22 | 45 | (85) | 19 | (46) | <0.001 |
|  | 1 | 17 | (18) | 10 | (14) | 7 | (29) |  | 4 | (7.5) | 13 | (32) |  |
|  | 2 | 11 | (12) | 7 | (10) | 4 | (17) |  | 4 | (7.5) | 7 | (17) |  |
|  | 3 | 2 | (2.1) | 2 | (2.9) | 0 | (0) |  | 0 | (0) | 2 | (4.9) |  |
| M-stage | 0 | 90 | (96) | 69 | (99) | 21 | (88) | 0.050 | 53 | (100) | 37 | (90) | 0.033 |
|  | 1 | 4 | (4.3) | 1 | (1.4) | 3 | (13) |  | 0 | (0) | 4 | (9.8) |  |
| p16 | No overexpression | 91 | (97) | 70 | (100) | 21 | (88) | 0.015 | 53 | (100) | 38 | (93) | 0.080 |
|  | Overexpression | 3 | (3.2) | 0 | (0) | 3 | (13) |  | 0 | (0) | 3 | (7.3) |  |
| p53 | 0-mutant type | 19 | (20) | 14 | (20) | 5 | (21) | 0.95 | 9 | (17) | 10 | (24) | 0.67 |
|  | Wild-type | 26 | (28) | 20 | (29) | 6 | (25) |  | 15 | (29) | 11 | (27) |  |
|  | Mutant-type | 49 | (52) | 36 | (51) | 13 | (54) |  | 29 | (55) | 20 | (49) |  |
| pRb | Loss | 19 | (20) | 13 | (19) | 6 | (25) | 0.56 | 8 | (15) | 11 | (27) | 0.20 |
|  | Positive | 75 | (80) | 57 | (81) | 18 | (75) |  | 45 | (85) | 30 | (73) |  |
| DFS: disease free survival; OS: overall survival; OSCC: oral squamous cell carcinoma; IQR: inter quartile range | | | | | | | | | | | | | |
